# Incidence of sexual dysfunction: a prospective survey in Ghanaian females

**DOI:** 10.1186/1477-7827-8-106

**Published:** 2010-09-01

**Authors:** Nafiu Amidu, William KBA Owiredu, Eric Woode, Otchere Addai-Mensah, Lawrence Quaye, Abass Alhassan, Edmond A Tagoe

**Affiliations:** 1 Department of Medical Laboratory Technology, Faculty of Allied Health Sciences, College of Health Sciences, Kwame Nkrumah University of Science and Technology, Kumasi, Ghana; 2 Department of Molecular Medicine, School of Medical Sciences, College of Health Sciences, Kwame Nkrumah University of Science and Technology, Kumasi, Ghana; 3 Department of Pharmacology, Faculty of Pharmacy and Pharmaceutical Science, College of Health Sciences, Kwame Nkrumah University of Science and Technology, Kumasi, Ghana; 4 Laboratory Department, Kwame Nkrumah University of Science and Technology Hospital, Kumasi, Ghana; 5 Department of Obstetrics and Gynaecology, Kwame Nkrumah University of Science and Technology Hospital, Kumasi, Ghana

## Abstract

**Background:**

Sexuality is a complex phenomenon that is being influenced by psychological as well as physiological factors. Its dysfunction includes desire, arousal, orgasmic and sex pain disorders. The present study aimed to assess the incidence of sexual dysfunction (SD) and related risk factors in a cohort of Ghanaian women.

**Method:**

The Golombok Rust Inventory of Sexual Satisfaction (GRISS) was administered to 400 healthy women between 18 and 58 years old (mean +/- SD: 30.1 +/- 7.9) domiciled in the Kumasi metropolis.

**Results:**

The response rate was 75.3% after 99 were excluded. Of the remaining 301 women, 50% were engaged in exercise, 26.7% indulge in alcoholic beverages and only 2% were smokers. A total of 62.1% of the women had attained high education, whilst, 28.9% were married. After logistic regression analysis, alcohol emerged (OR: 2.0; CI: 1.0 - 3.8; p = 0.04) as the main risk factor for SD. The overall prevalence of SD in these subjects was 72.8%. Severe difficulties with sexual function were identified in 3.3% of the studied population. The most prevalent areas of difficulty were anorgasmia (72.4%), sexual infrequency (71.4%), dissatisfaction (77.7%), vaginismus (68.1%), avoidance of sexual intercourse (62.5%), non-sensuality (61.5%) and non-communication (54.2%). Whereas 8% had severe difficulties with anorgasmia, only 6% had severe difficulties with vaginismus.

**Conclusion:**

SD affects more than 70% of Ghanaian women who are sexually active. Alcohol significantly influences sexual activity.

## Background

Human sexuality is a complex process which is coordinated by the neurologic, vascular and endocrine systems [1]. Sexuality does not only include family, societal and religious beliefs, it can also be influenced by aging, health status and personal experience as well as socio-economic status. Sexual dysfunction (SD) is an important public health problem that is more prevalent in women than in men [2]. Previous studies have established that up to about 76% of women experience some form of SD - that is, a sexual problem that they find distressing [3].

The study and knowledge of SD has been largely unexplored and its incidence is said to vary widely possibly due to differences in criteria for defining SD: the population involved, cultural background, socio-economic level, quality of psychosexual relationships and income. An important development has been the classification of SD by the American Psychiatric Association documented in the Diagnostic and Statistical Manual of Mental Disorder (DSM-IV) [4]. The Diagnostic and Statistical Manual of Mental Disorders, 4th edition, text revision (DSM-IV-TR) identifies 6 female sexual dysfunctions: hypoactive sexual desire disorder, sexual aversion disorder, sexual arousal disorder, orgasmic disorder, dyspareunia and vaginismus (i.e. sexual pain disorders). Using these criteria, Laumann *et al.*, found the prevalence of SD to be 43% in a survey of 1749 women aged 18 to 59 years in the United States [5]. SD was found to vary according to the physical and psychic health and ethnic group in this study [5]. In view of the reasons expressed above, the aim of this work was to study the incidence and type of sexual dysfunction in healthy sexually active Ghanaian women with a steady heterosexual relationship, and related risk factors.

## Methods

### Subjects

Four hundred (400) Ghanaian women were randomly sampled in a cross-sectional study in the Kumasi metropolis, Ghana between December 2009 and May 2010. All the women reported a steady heterosexual relationship with one or more partners for more than one year. No woman had been a drug abuser or had surgical interventions which might modify sexual behaviour. Demographic and socio-cultural variables such as age, marital status, years of scholarship, smoking status, level of exercise and alcohol intake were recorded. Exercise was defined as any activity causing light perspiration or a slight to moderate increase in breathing or heart rate for at least 30 minutes. Alcohol intake was defined as the intake of at least one bottle of an alcoholic beverage per week. Regarding smoking, individuals were classified as smokers based on whether the respondent is in the habit of smoking at least one cigarette a day. Participation of the respondents was voluntary and informed consent was obtained from each participant. The study was approved by the Committee on Human Research, Publication and Ethics of the School of Medical Science and the Komfo Anokye Teaching Hospital, Kumasi.

### Sexual function

This was assessed by the Golombok Rust Inventory of Sexual Satisfaction (GRISS). One of the main reasons for choosing the GRISS is its simplicity and clarity of the assessment of results. The GRISS has 28 items on a single sheet and it is used for assessing the existence and severity of sexual problems. All the 28 questions are answered on a five-point scale from "always", through "usually', "sometimes", and "hardly ever", to "never". Responses are summed up to give a total raw score (range 28-140). In addition, seven subscale scores are derived (i.e. vaginismus, anorgasmia, avoidance, non-sensuality, non-communication, infrequency and dissatisfaction). The total score and subscale scores are transformed using a standard nine point scale, with high scores indicating greater problems. Scores of five or more are considered to indicate sexual dysfunction. The reliability of the overall scales has been found to be 0.87 for women, and that of the subscales on average 0.74 (ranging between 0.61 and 0.83). Validity has been demonstrated under a variety of circumstances [6-8].

### Statistical analysis

The data were presented as mean ± SD or percentages. Continuous data were analyzed using unpaired *t*-tests whilst categorical variables were analyzed using Fischer's exact test or chi square for trend test. Logistic regression was used to assess the simultaneous influence of different variables in sexuality. In all statistical tests, a value of *p *< 0.05 was considered significant. The entry of the variables into the model was considered if p value is less than 0.05, and a stepwise procedure was applied. All analysis were performed using SigmaPlot for Windows, Version 11.0, (Systat Software, Inc. Germany) [9]

## Results

Of the initial 400 women recruited, 99 were excluded: 11 had severe chronic disease, 16 denied participations, 31 had difficulties in understanding the survey and the questionnaires from 41 women were incomplete, leaving 301 complete and evaluable questionnaires, indicating a response rate of 75.3%. The mean age of the participating women was 30.1 ± 7.9 years. The median age was 28.0 years with inter-quartile range of 25.0 to 34.0 and age range of 18 to 58 years. Almost 50% of the women were engaged in exercise, 26.7% indulge in alcoholic beverages and only 2% were smokers. A total of 62.1% of the women had attained high education, whilst 28.9% were married. About 18% of the participating women were 36 years or older.

The effect of different socio-demographic variables on the SD risk is recorded in Table 1. The only significant factor from this study that increased female SD as determined by univariate analysis was alcohol (OR: 2.0; CI: 1.1 - 3.9; p = 0.03). None of the other factors modified SD risk significantly (Table 1). After logistic regression analysis, alcohol still emerged (OR: 2.0; CI: 1.0 - 3.8; p = 0.04) as the main risk factor for SD.

**Table 1:** Univariate analysis of risk factors for female sexual dysfunction

Variables	n/N*	Rate of SD (%)	OR(CI 95%)	P value
***Exercise***				
Yes	109/149	73.2	1.0(0.6-1.7)	0.9
No	107/150	71.3		
***Alcohol***				
Yes	66/80	82.5	2.0(1.1-3.9)	0.03
No	151/220	68.6		
***Smoking***				
Yes	6/6	100.0	1.0(0.3-90.7)	0.2
No	212/294	72.1		
***Married***				
Yes	84/87	96.6	1.2(0.7-2.1)	0.6
No	135/214	63.1		
***Educational attainment***				
High	131/187	70.1	1.6(0.9-2.7)	0.1
Low	88/114	77.2		
***Age ≥ 36 years***				
Yes	37/53	69.8	1.3(0.7-2.5)	0.4
No	182/247	73.7		

*Number of subjects with SD/number of subjects in each category.

**Figure 1:**
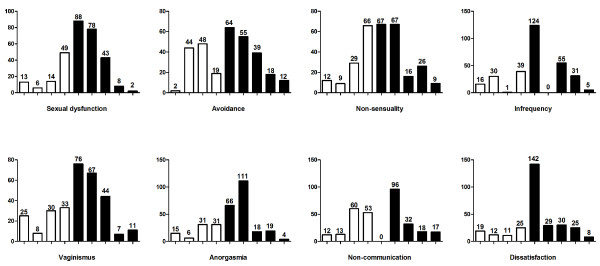
**Participants' sexual function scores for each GRISS subscale (n = 301)**. Each graph shows the distribution of scores (from 1 to 9 on the x-axis) for each GRISS subscale, with the number of women (y-axis) above each score. Normal scores are 1 to 4 (*clear columns*) and abnormal scores are 5 to 9 (*black columns*).

Most of the women (219 out of 301; 72.8%) had one or more subscale scores reflecting sexual problems (score of 5 or above). The most prevalent areas of sexual difficulty (Figure 1) were dissatisfaction (234 out of 301; 77.7%), anorgasmia (218 out of 301; 72.4%), sexual infrequency (215 out of 301; 71.4%), vaginismus (205 out of 301; 68.1%), avoidance, of sexual intercourse (188 out of 301; 62.5%), and non-sensuality (185 out of 301; 61.5%). The levels of communication scores were relatively high, with only 54.2% of the participants having a score indicating non-communication. However, 3.3% (i.e. 10 out of 301) of the studied population had severe SD. About 12% had severe difficulties with sensuality, frequency and communication. Eleven percent (11%) and 10% had severe difficulties with satisfaction and avoidance respectively. Whereas 8% had severe difficulties with anorgasmia, only 6% had severe difficulties with vaginismus (Figure 1).

When the study population was stratified by age, the prevalence of SD generally remained stable (i.e. around 73%). A marked decrease and increase in the prevalence of SD is however noted in the 38 to 42 years group and ≥ 48 year groups respectively. Out of the seven subscales, only vaginismus was observed to significantly increase with age (Table 2).

**Table 2:** Prevalence of sexual dysfunction according to age group using GRISS

	Age (years)							
**Variable**	**18-22**	**23-27**	**28-32**	**33-37**	**38-42**	**43-47**	**≥ 48**	**P value**
***n***	***38***	***94***	***78***	***41***	***21***	***13***	***14***	

Difficulties (%)*								
Sexual dysfunction	73.7	72.3	73.1	73.2	57.1	76.9	85.7	0.9
Avoidance	76.3	64.9	57.7	56.1	47.6	92.3	57.1	0.3
Non-sensuality	52.6	61.7	65.4	68.3	47.6	69.2	50.0	1.0
Infrequency	65.8	69.1	80.8	75.6	52.4	61.5	71.4	0.8
Vaginismus	65.8	63.8	66.7	65.9	81.0	76.9	85.7	0.04
Anorgasmia	71.2	70.2	74.4	68.3	66.7	92.3	85.7	0.2
Non-communication	52.6	56.4	52.6	48.8	71.4	53.8	42.9	0.9
Dissatisfaction	71.1	74.5	84.6	80.5	76.2	76.9	71.4	0.7
Severe difficulties (%)**								
Sexual dysfunction	2.6	5.3	2.6	4.9	0.0	0.0	0.0	0.3
Avoidance	21.1	12.8	5.1	2.4	4.8	7.7	21.4	0.2
Non-sensuality	10.5	14.9	11.5	14.6	4.8	0.0	7.1	0.2
Infrequency	13.2	11.7	11.5	7.3	14.3	15.4	21.4	0.6
Vaginismus	0.0	8.5	2.6	9.8	4.8	7.7	14.3	0.2
Anorgasmia	5.3	9.6	10.3	7.3	0.0	0.0	7.1	0.4
Non-communication	13.2	14.9	9.0	14.6	9.5	7.7	0.0	0.2
Dissatisfaction	10.5	13.8	9.0	14.6	9.5	0.0	7.1	0.3

*Score of 5-9 and **Score of 8 or 9. P values were derived from chi square for trend.

SD correlated with non-sensuality, vaginismus, anorgasmia and dissatisfaction with a large size effect. As can be seen in Table 3, most of the areas of sexual function were related, with small to large effect sizes [10]. For the purpose of interpretation, Cohen [10] considered 0.10 <*r <*0.30 as small, 0.30 <*r <*0.50 as medium and r > 0.50 as large. Women with sexual dissatisfaction were also more likely to have problems with all the other subscales apart from avoidance (r = 0.07). Women with anorgasmia were more likely to have avoidance of sexual activity (*r = 0.13*, *P *< 0.05), non-sensuality (r = 0.42, P < 0.0001), decreased frequency of sexual activity (r = 0.21, P < 0.0001) and vaginismus (*r *= 0.44, *P *< 0.0001). Vaginismus also increased with increased non-sensuality (r = 0.37, P < 0.0001) and infrequency (r = 0.12, P < 0.05) (Table 3).

**Table 3:** Pearson Product Moment Correlation Coefficient Between Female Sexual Dysfunction Including the 7 Subscales of the GRISS *(N = *301)

Variables	SD	AV	NS	INF	VAG	ANG	NC	DIS
Age	0.04	-0.12*	0.00	0.05	0.15**	0.09	-0.02	0.05
Sexual dysfunction (SD)		0.30***	0.67***	0.36***	0.62***	0.70***	0.32***	0.62***
Avoidance (AV)			-0.03	-0.09	0.01	0.13*	0.07	0.07
Non-sensuality (NS)				0.17**	0.37***	0.42***	0.14*	0.30***
Infrequency (INF)					0.12*	0.21***	0.14*	0.21***
Vaginismus (VAG)						0.44***	0.12*	0.27***
Anorgasmia (ANG)							0.10	0.31***
Non-communication (NC)								0.16**

*Correlation is significant at the 0.05 level (2-tailed), **Correlation is significant at the 0.01 level (2-tailed), ***Correlation is significant at the 0.001 level (2-tailed). Boldface r = Pearson product moment correlation coefficient with a medium size (0.30 ≤ r ≥ 0.50) effect: boldface and italiced r = Pearson product moment correlation coefficient with a large size (r > 0.50) effect, DIS = dissatisfaction.

## Discussion

It is noteworthy that a sexual problem is perceived as a disorder only if a woman perceives it to be so, with impaired sexual desire as the most common presentation [11]. Sexual problems in women may contribute to infertility, emotional burden, health burden and a burden on the relationship. Emotional burdens may lead to a sense of embarrassment and awkwardness because of difficulty in satisfying the partner.

In assessing six socio-demographic factors (exercise, alcohol, smoking, marital status, educational status and age) as risk factors for female sexual dysfunction, alcohol intake was the sole significant risk factor for the development of sexual dysfunction in this study. Female respondents who took alcoholic beverages were 2 times at risk of developing sexual dysfunction than those who were not taking alcoholic beverages. Alcohol is known to reduce genital response [12,13] and this might be a reason for the significant increase in the rate of sexual dysfunction observed in the study respondents taking alcoholic beverages. It is also possible that women who consume alcoholic beverages in connection with sexual activities may initially have sexual function problems and, therefore, consume alcohol in order to reduce anxiety which might further increase the risk of sexual dysfunction.

A study conducted by Amidu *et al.*, (under review) on Ghanaian perceptions on intravaginal ejaculatory latencies established that, women, desire that intercourse lasts about twice (8 - 19 minutes) as long as what sex therapists consider to be adequate (3 - 7 minutes). A sexual performance level below this perceived duration may therefore lead to distress and displeasure which ultimately results in the use of varied remedies to restore performance levels. Patronage of aphrodisiacs which are mostly formulated in alcoholic base in Ghana is on the ascendancy (personal observation) and their chronic usage may increase the risk of sexual dysfunction as established in this study. The other socio-demographic variables when assessed as risk factors for female sexual dysfunction did not contribute significantly to the rate of developing sexual dysfunction from this study.

The prevalence rate of sexual dysfunction was 73% in females in this study and this finding is consistent with that of Frank *et al.*, [14] and Spector *et al.*, [3] who reported prevalence rates of 76% in women. Such a high rate of sexual dysfunction may compromise the health status and quality of life of women due to a general sense of embarrassment, awkwardness and the inability to satisfy their partners fully. The most common female SD problems in this study were sexual dissatisfaction (77.7%), anorgasmia (72.4%) and sexual infrequency (71.4%). Jindal *et al.*, [15] in an evaluation of 200 Indian women reported sexual infrequency and anorgasmia as the most common problems.

Sexual dissatisfaction could be multifactorial in nature and the varied causes were not explored by this prospective survey. However, the high perception of women on the length of ejaculatory latencies coupled with a reported premature-like ejaculation prevalence of 64.7% in Ghanaian men (Amidu *et al.*, under review) could play a significant role in the observation that women do not achieve adequate satisfaction and gratification from their partners during sexual intercourse. A general lack or reduced sexual satisfaction could thus lead to anorgasmia and sexual infrequencies as observed in the percentage prevalence. Sexual trauma resulting from rape is a significant contributing factor for the high incidence of anorgasmia. Sexual trauma results in increased risk of experiencing arousal disorders and this induces lasting psychosocial disturbances which ultimately affects sexual function and its related orgasmic disorders [16]. Sexual infrequency may be a problem in its own right and may possibly be influenced by both health related and psychosocial factors [5]. Stress induced events as a result of psychosocial factors and interplay of a host of other factors not explored by this study may reduce opportunities for sexual interaction [5].

The prevalence of vaginismus from this study was 68.1% and this is consistent with the finding of Tayebi *et al.*, [17] which reported a percentage of 67.7. The prevalence rates of the other subscales are: avoidance of intercourse (62.5%), non-sensuality (61.5%) and non-communication (54.2%).

The high prevalence of SD observed in the ≥ 48 year group may be attributed mostly to vaginismus which showed a significantly high prevalence in comparison to the other areas of sexual difficulty and a positive linear relationship with a small size effect to age. Vaginismus would therefore tend to increase with increasing age. Laumann *et al.*, [5] in his study findings, stated that prevalence of sexual problems decrease with increasing age except for those who have some difficulties with lubrication. Lubrication is required for smooth and enjoyable sexual intercourse and as such inadequate lubrication is associated with vaginismus in the ≥ 48 year group. Oberg *et al.*, [18] in a study on women aged 18-65 found vaginismus to be strongly associated with sexual distress.

Furthermore, vaginismus had a small size linear effect with sexual infrequency and a large sized linear effect with non-sensuality. This means that respondents in the ≥ 48 year group will often tend to have infrequent sexual activities and waning sexual desire as a result of vaginismus. Hayes *et al.*, [19] reported a linear decrease in desire with increasing age from 20 to 70 and Abdo *et al.*, [20] reported a general negative effect of age on desire and a positive effect on pain and these findings are consistent with that of this study.

Non-communication showed the least prevalence amongst the areas of sexual difficulty and correlated linearly to a small size effect with dissatisfaction, vaginismus, sexual infrequency and non-sensuality. Decreased partner interaction and lack of communication in the relationship will therefore most certainly be linked with lack of desire, sexual infrequency, sexual dissatisfaction and vaginismus. Byers, [21] showed intimate communication to be associated with changes in both relationship and sexual satisfaction over a time period of 18 months. Others reporting on poor communication also reported a decrease in both relationship and sexual satisfaction during this time period while those reporting good communication reported an increase.

King *et al.*, [22] reported that having one SD is commonly associated with a secondary SD. Significant co-morbidities were observed between the subscales of SD assessed in this study. Sexual dissatisfaction correlated with non-sensuality and anorgasmia on a large size scale and sexual infrequency, vaginismus and non-communication on a small size scale. Sexual dissatisfaction often leads to loss of sensuality and subsequent orgasmic disorders which results in ripple effects of sexual infrequencies, sexual pains and a general lack of communication in the relationship [19,23].

Non-communication on the other hand correlated on a small size scale with non-sensuality, sexual infrequency and vaginismus. Poor communication in a relationship may lead to reduced sexual desire, infrequent sexual episodes and a resultant sexual pain. Anorgasmia correlated with non-sensuality and vaginismus to a large size effect whereas infrequency and avoidance correlated on a small size effect. Reduced sensuality for a partner wanes desire for sexual intercourse which culminates in orgasmic disorders and pain during sex [24]. Such episodes may lead to sexual infrequencies and a general avoidance of sexual intercourse with the partner. Vaginismus correlated on a large size effect with non-sensuality and on a small size effect with sexual infrequency. Women will therefore tend to have vaginismus when there is reduced sensuality which could further lead to sexual infrequency. Sexual infrequency correlated with non-sensuality on a small size effect and certainly so, lack of desire for a partner in a relationship could lead to infrequent sexual acts. It is, however, worthy to note that such existent correlations do not imply causality but it is reasonable to assume that one variable could result in the other, e.g., suffering from sexual pain will result in sexual dissatisfaction and on the other hand being dissatisfied and anxious about sexual activities or one's sexuality might also result in pain.

## Conclusion

In an overall analysis, sexual dissatisfaction had the highest prevalence and the highest co-morbidity with the other risk factors of SD making it a worthy risk factor to investigate and addressed thoroughly as a totality of such risk factors culminating in SD could lead to low feelings of physical and emotional satisfaction and low feelings of happiness and a general diminished quality of life in women. Alcohol is a significant risk factor for developing sexual dysfunction.

## Competing interests

The authors declare that they have no competing interests.

## Authors' contributions

NA, WKBAO and EW developed the concept and designed the study. NA, WKBAO, EW, OA-M, LQ, AA and EAT administered the questionnaire, analysed and interpreted the data. NA, OA-M, LQ, AA and EAT drafted the manuscript. NA, WKBAO, EW, OA-M, LQ, AA and EAT revised the manuscript for intellectual content. All authors read and approved the final manuscript.
